# Utilizing Human-Induced Pluripotent Stem Cells to Study Cardiac Electroporation Pulsed-Field Ablation

**DOI:** 10.1161/CIRCEP.123.012278

**Published:** 2024-02-12

**Authors:** Leonid Maizels, Eyal Heller, Michal Landesberg, Shany Glatstein, Irit Huber, Gil Arbel, Amira Gepstein, Doron Aronson, Shirley Sharabi, Roy Beinart, Amit Segev, Elad Maor, Lior Gepstein

**Affiliations:** 1 Division of Cardiology, Leviev Center of Cardiovascular Medicine, Sheba Medical Center, Ramt Gan, Israel (L.M., E.H., R.B., A.S., E.M.).; 2 Faculty of Medicine, Tel-Aviv University, Tel Aviv-Yafo, Israel (L.M., R.B., A.S., E.M.).; 3 Talpiot Sheba Medical Leadership Program, Sheba Medical Center, Ramat Gan, Israel (L.M., E.M.).; 4 Department of Cardiology, Royal Melbourne Hospital, Australia (L.M.).; 5 Sohnis Laboratory for Cardiac Electrophysiology and Regenerative Medicine, Rappaport Faculty of Medicine, Technion, Haifa, Israel (M.L., S.G., I.H., G.A., A.G., L.G.).; 6 Division of Cardiology, Rambam Health Care Campus, Haifa, Israel (D.A., L.G.).; 7 Advanced Technology Center and Department of Radiology, Sheba Medical Center, Ramat Gan, Israel (S.S.).

**Keywords:** arrhythmia, cardiac, electroporation, human induced pluripotent stem cell, voltage-sensitive dye imaging

## Abstract

**BACKGROUND:**

Electroporation is a promising nonthermal ablation method for cardiac arrhythmia treatment. Although initial clinical studies found electroporation pulsed-field ablation (PFA) both safe and efficacious, there are significant knowledge gaps concerning the mechanistic nature and electrophysiological consequences of cardiomyocyte electroporation, contributed by the paucity of suitable human in vitro models. Here, we aimed to establish and characterize a functional in vitro model based on human-induced pluripotent stem cells (hiPSCs)-derived cardiac tissue, and to study the fundamentals of cardiac PFA.

**METHODS:**

hiPSC-derived cardiomyocytes were seeded as circular cell sheets and subjected to different PFA protocols. Detailed optical mapping, cellular, and molecular characterizations were performed to study PFA mechanisms and electrophysiological outcomes.

**RESULTS:**

PFA generated electrically silenced lesions within the hiPSC-derived cardiac circular cell sheets, resulting in areas of conduction block. Both reversible and irreversible electroporation components were identified. Significant electroporation reversibility was documented within 5 to 15-minutes post-PFA. Irreversibly electroporated regions persisted at 24-hours post-PFA. Per single pulse, high-frequency PFA was less efficacious than standard (monophasic) PFA, whereas increasing pulse-number augmented lesion size and diminished reversible electroporation. PFA augmentation could also be achieved by increasing extracellular Ca^2+^ levels. Flow-cytometry experiments revealed that regulated cell death played an important role following PFA. Assessing for PFA antiarrhythmic properties, sustainable lines of conduction block could be generated using PFA, which could either terminate or isolate arrhythmic activity in the hiPSC-derived cardiac circular cell sheets.

**CONCLUSIONS:**

Cardiac electroporation may be studied using hiPSC-derived cardiac tissue, providing novel insights into PFA temporal and electrophysiological characteristics, facilitating electroporation protocol optimization, screening for potential PFA-sensitizers, and investigating the mechanistic nature of PFA antiarrhythmic properties.

**WHAT IS KNOWN?**

- Pulsed-field ablation (PFA) is emerging as a nonthermal ablation modality for arrhythmia treatment.
- Although initial clinical studies found PFA both safe and efficacious, there are significant knowledge gaps concerning the mechanistic nature and electrophysiological consequences of cardiomyocyte electroporation, contributed by the paucity of suitable human in vitro models

**WHAT THE STUDY ADDS**

- We used an in vitro hiPSC-based cardiac tissue model to provide functional insights into PFA mechanisms and electrophysiological consequences.
- PFA induced short- and long-term electrophysiological silencing, stemming from acute cellular changes (membrane depolarization and increased intracellular Ca^2+^), cellular damage, and regulated cell death. Both reversible and irreversible electroporation components were identified, highlighting that the acute presence of functional conduction blocks does not necessarily mean long-term lesion persistence, and that further research is required to identify robust predictors for long-term PFA persistence.
- We compared monophasic and high-frequency-PFA protocols, demonstrating significant differences in the electrophysiological outcomes, including electroporation irreversibility thresholds. Hence, protocol advantages, disadvantages and temporal kinetics should be further studied, characterized, and standardized for effective clinical use.
- A novel approach to enhance cardiac PFA effects was demonstrated through electroporation Ca^2+^-sensitization, which may allow for further clinical protocol optimization.
- The ability to terminate arrhythmias with PFA was shown through the formation of sustainable bidirectional conduction line blocks, while deploying ablation lesions with high-spatial precision.

Catheter ablation is the treatment of choice for a growing number of cardiac arrhythmias. Current ablative approaches are primarily based on thermal energy sources and may, therefore, be limited by collateral tissue damage, blood clotting, procedural time, and tissue penetrance.^1–3^ Electroporation is a biophysical phenomenon of increased cell-membrane permeability following pulsed electric fields application. Electroporation is a graded and complex phenomenon. It may be transient, with the cellular membrane subsequently resealing (reversible electroporation), which may be used for drug or gene delivery while maintaining cell viability. When the cells are exposed to field intensities above a certain threshold, the increased membrane permeability coupled with further dysregulation of multiple cellular processes does not allow for cellular recovery, culminating in cell death (irreversible electroporation).^1,4–7^ Employing pulsed electric fields for cardiac ablation (pulsed-field ablation, PFA) may present significant advantages over the currently used thermal approaches, and subsequently the clinical interest in PFA as a nonthermal and potentially more cardio-selective ablation modality is rapidly growing.^1,8–14^ Although preclinical work and recent clinical studies found PFA to be both safe and efficacious,^8–10,15–17^ there are significant knowledge gaps concerning cardiac PFA, partially due to paucity of suitable human in vitro cardiac tissue models. Beyond the fact that currently used PFA protocols are not fully disclosed by the industry and the emerging concerns regarding coronary spasm/stenosis^18,19^ or phrenic nerve injury,^16^ there are many additional aspects requiring further study, including: PFA delivery protocols and equipment optimization, electroporation reversibility thresholds and time constants, tissue selectivity, and possible PFA modifiers or sensitizers, PFA lesion characteristic and their long-term sustainability, the characterization of PFA-mediated cellular damage, and the mechanistic nature of PFA antiarrhythmic properties. Addressing these issues is crucial for further expansion of PFA in the cardiac clinical setup.

The human-induced pluripotent stem cell (hiPSC) technology allows reprogramming of patient-specific somatic cells into pluripotent stem cells, that can be coaxed to differentiate into cardiomyocytes.^20,21^ In the cardiac field, we and others have established patient/disease-specific hiPSC-derived cardiomyocyte (hiPSC-CM) models from healthy individuals and from patients inflicted with different cardiac arrhythmogenic syndromes.^22–26^

Moving from the cellular level to more complex 2- and 3-dimensional models, the use of advanced optical-mapping techniques^27–30^ allowed the detailed study of cardiac electrophysiological properties using hiPSC-CMs. This included characterization of conduction and repolarization properties, arrhythmogenic susceptibility, the mechanisms underlying reentrant arrhythmia induction and perpetuation, and the effects of antiarrhythmic pharmacological and nonpharmacological interventions.^27–30^ With the advancements made in the differentiation and maturation of hiPSC-CMs, it has been consistently shown that hiPSCs may be used to model primary human cardiomyocytes owing to their similar electrophysiological, molecular, structural, and mechanical properties.^31^ Although of clinical and scientific relevance, it is noteworthy that nonhuman or heterologous expression system models may differ significantly from human cardiac tissue in their expression profile of ion channels, calcium-handling proteins, and other structural and functional proteins.^32^ Recently, hiPSC-based cellular models were used to study PFA cardioselectivity and irreversibility thresholds using cell-viability markers, not addressing however electrophysiological parameters.^13,33^ In the current study, we aimed to harness the advancements made in using hiPSC-derived cardiac tissue models in electrophysiology research to study the fundamentals of cardiomyocyte electroporation, with a specific focus on the impact of PFA on the functional and electrophysiological properties of these models.

## METHODS

The data that support the findings of this study are available from the corresponding authors upon reasonable request.

### Cardiomyocyte Differentiation and Generation of hiPSC-Derived Cardiac Cell Sheets

Cardiomyocyte differentiation of a previously established healthy-control hiPSC line^22^ was achieved using the monolayer-directed differentiation system as previously described.^27,29^ The generation and use of the hiPSC lines and their differentiated cardiomyocytes was approved by the institutional review board (Helsinki) committee of the Rambam Medical Center (0170-019-RMB), and subjects gave their informed consent for hiPSC generation and use. To generate hiPSC-derived cardiac circular cell sheets (hiPSC-CCSs), differentiating beating monolayers (14–60 days) were enzymatically dissociated and seeded as circular cell sheets (≈0.5–1.5 cm diameter) on Matrigel-coated culture plates, at a seeding density of 0.5 to 1.5×10^6^ cells in 150 μL drops, similar to previous reports.^27,28^ Tissues were cultured in RPMI/B27 containing 1% penicillin/streptomycin and blebbistatin (5 μmol/L).

### PFA Experiments

**Figure 1.**
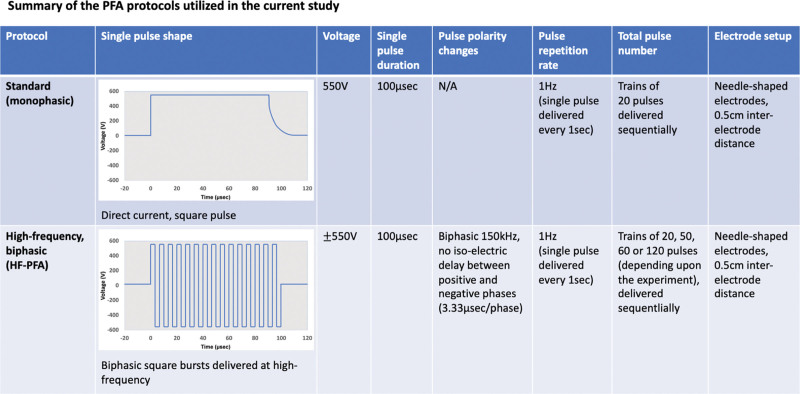
**Summary of the pulsed-field ablation (PFA) protocols used in the current study.** Standard (monophasic) and high-frequency (HF) biphasic PFA (HF-PFA) protocols were used and compared. Monophasic pulses were delivered as square pulses of 550 V, and 100 µsec duration per single pulse. Trains of 20 pulses were delivered sequentially at 1 Hz frequency (1 pulse delivered each second), via 2 needle-shaped electrodes with 0.5 cm inter-electrode spacing. HF biphasic pulses were delivered as biphasic bursts of ±550 V, and 100 µsec duration per single pulse, with 150 kHz biphasic frequency, and no isoelectric delay between phases (hence each single pulse consisted of 15 biphasic bursts, with 3.33 µsec positive and negative phases occurring interchangeably). Depending upon the experiment, trains of 20, 50, 60, or 120 pulses were delivered sequentially at 1 Hz frequency (1 pulse delivered each second) via 2 needle-shaped electrodes with 0.5 cm inter-electrode spacing.

Pulsed fields were delivered using a standard electroporation pulse generator (BTX-ECM830, Holliston, MA) or a custom-made generator allowing the delivery of high-frequency, high-voltage protocols.^34^ Pulses were delivered via 2 needle-shaped electrodes (BTX-450168, 0.5 cm inter-electrode distance), by direct contact with the hiPSC-CCSs. The detailed PFA protocols are described in Figure 1.

PFA was delivered as a train of 20, 40, 50, 60, or 120 sequential pulses, depending upon the specific experiment. The waveform used was either monophasic or high-frequency biphasic, as specified in each experiment. Each single pulse duration was 100 μsec, and pulses were delivered at 1 Hz repetition frequency (Figure 1). In experiments incorporating point by point ablation, pulse trains were delivered in consecutive locations across the culture midline. Treatment protocols were randomly assigned to hiPSC-CCSs derived from different batches and differentiation passages, similar to previous reports.^27–29^

### Optical Mapping

The hiPSC-CCSs were loaded with either FluoVolt or Fluo-4 as described.^27–29^ ArcLight-expressing hiPSC-CCSs were also used for voltage mapping.^27–29^ The health of the preparations was assessed functionally by documenting typical electric activity with synchronous and homogeneous conduction in the constructed activation maps, by the ability to pace the cultures at 1 Hz, and by the macroscopic and microscopic appearance of the hiPSC-CCSs. Signal recordings were performed using a high-speed EM-CCD camera mounted on a fluorescent macroscope (260 frames/s, 4×4 binning^27–29^). Data were analyzed using custom-written software (including OMProCCD, curtesy of Prof. Bum-Rak Choi) for generation of activation, conduction, and phase maps as described.^27–29^ For identification of PFA-induced electrically silenced areas/lesions, a custom-written algorithm based on frequency analysis in each pixel was used. Lesion-area measurements were performed using ImageJ software. Phase-maps were created using ElectroMap software.^35^ Experiments were performed at 37°C in tyrode solution, containing: NaCl 140 mmol/L; KCl 5.4 mmol/L; CaCl_2_ 1.8 mmol/L; MgCl_2_ 1.0 mmol/L; HEPES 10 mmol/L; glucose 10 mmol/L (pH 7.4 with NaOH), and blebbistatin (5 μmol/L). Ca^2+^ PFA sensitization was evaluated in Tyrode’s containing 4 mmol/L CaCl_2_.

### Finite Element Modeling

To calculate the electric field distribution within the hiPSC-CCSs, a 2-dimensional finite elements model^36^ was constructed using COMSOL Multiphysics 5.3a (COMSOL Multiphysics; Stockholm, Sweden). The 2-dimensional geometry was designed to match the dimensions of the experimental setup. The well was modeled as a 2×2 cm^2^ to match the macroscope’s field of view. The hiPSC-CCS was modeled as a circle (0.8 cm radius) at the center of the well and the stainless steel electrodes were modeled as 2 circles (0.05 cm radius) positioned 0.5 cm apart. The medium electric conductivity^13^ was set at 2.3 S/m and the electrodes conductivity was set to 1.7×10^6^ S/m. The electric field was described by the Laplace equation for electric potential distribution in a volume conductor: (1) ∇⋅(σ∇φ)=0, where σ is the electric conductivity of the culture, and φ is the potential. Dirichlet boundary condition was applied to the surface of the electrode: (2) $\varphi =\varphi_{0}$ and to the ground (3) φ=0, where $\varphi_{0}$ is the applied potential on the active electrode. The boundaries where the analyzed domain was not in contact with an electrode were treated as electrically isolative and Neumann boundary condition was set to zero on the outer border of the model: (4) $\frac{\partial \varphi }{\partial n}=0$, where *n* denotes the normal to the boundary and φ is the potential.

To calculate the electric field thresholds, the lesion areas as derived from optical mapping images were correlated with the electric field contours using custom-written MATLAB software (MathWorks, Inc, MA).

### Flow-Cytometry Experiments

MEBCYTO apoptosis kit (MBL) was used to evaluate cell death. Propidium iodine (PI) is a cell-death marker that binds DNA while penetrating dead or dying cells. Annexin-V binds to phosphatidylserine, which is translocated to the outer membrane portion of cells undergoing programmed/regulated-death. Hence, combining Annexin-V and PI staining may allow differentiating between accidental/necrotic and programmed/regulated cell-death mechanisms. Untreated control hiPSC-CCSs, and hiPSC-CCSs immediately (T_0_), and 24 hours following PFA were enzymatically dissociated, washed with PBS, resuspended in binding buffer, and loaded with PI, and Annexin-V-FITC (15 minutes). Over 50 000 cells were analyzed in each sample, using the LSR Fortessa flow cytometer (BD-Biosciences). Data were analyzed using FlowJo software.

### Statistical Analysis

PFA lesion areas are reported as mean±SD. Comparisons of cell-death markers in flow-cytometry experiments and of field intensity thresholds as derived from numerical modeling were performed using 1-way ANOVA, with Tukey’s post hoc (GraphPad, Prism9). To analyze lesion recovery over time, a repeated-measures linear mixed effect model with an unstructured covariance matrix was applied. The model included fixed factors for group (electroporation protocol), time, group-by-time interaction, and a random effect for the individual experiments to accommodate culture-specific deviations in intercept. As lesion-recovery slope changes were not constant, we applied a 2-piecewise random coefficients model allowing separation of an early rapid recovery period from a later component, each with different slopes. This knot value was selected by fitting the model over a range of potential knots and selecting the model that had the lowest Akaike Information Criteria. *P*<0.05 was considered statistically significant. Mixed modeling was performed using Stata version 17.0 (College Station, TX).

## RESULTS

### Standard (Monophasic) PFA in hiPSC-CCSs

**Figure 2.**
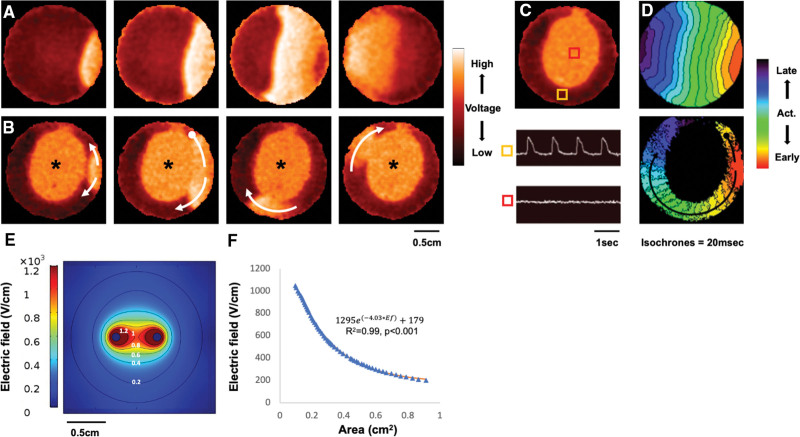
**Optical mapping of human-induced pluripotent stem cell (hiPSC)-derived cardiac circular cell sheets (hiPSC-CCSs). A**, Sequential fluorescent images (from left-to-right panels) taken from dynamic optical mapping display of FlouVolt-loaded hiPSC-CCS during point electric stimulation. Note the uniform depolarization wave (white) signal propagation from the pacing site. **B**, The same hiPSC-CCS following monophasic PFA (20 pulses). Note that the PFA lesion (asterisks) is relatively depolarized (white), electrically inactive, and leads to conduction block. Subsequently, the paced impulse now propagates around the lesion (white arrows). **C**, Optical signal traces from 2 different regions of interest (ROI) within the same hiPSC-CCS showing typical action-potentials from the unaffected tissue (yellow ROI) and no electric activity within the PFA lesion (red ROI). **D**, Activation maps of the same hiPSC-CCS at baseline (top) and post-PFA (bottom). Note uniform conduction at baseline and the formation of a conduction block following PFA, with signal propagation around the lesion (arrows), including an early conduction block in the counterclockwise direction (oval arrow-cap). Isochrones = 20 msec. **E**, Numerical modeling of the field intensity distribution within the hiPSC-CCSs while applying pulsed electric fields via 2 needle-shaped electrodes. Note that the highest field strength is formed around each of the electrodes and tappers down with distance. Also note the elliptical shape of the isointensity regions (isochrones=0.2 kV/cm), which correlates with the shape of the PFA lesions as recorded by optical mapping. **F**, The correlation between electric field intensities and the culture area exposed to each intensity could be fitted to an exponential function ($\left[1295e^{\left(-4.03*Ef\right)}+179\right]$, where Ef represents the electric field; R^2^=0.99, *P*<0.001).

We evaluated the electrophysiological effects of monophasic PFA (Figure 1) in the hiPSC-CCSs. Compared with baseline recordings (Figure 2A; Video S1), we found that PFA instantly generated electrically silenced lesions within the hiPSC-CCSs (Figure 2B;  Video S2), functioning as conduction barriers. Interestingly, beyond the lack of action-potential generation, PFA-affected areas were also characterized by a relatively depolarized resting membrane potential as compared with nonaffected areas, reflecting the disruption of membrane integrity and its normal electric function. Importantly, while conduction was blocked in the affected region, the surrounding tissue was electrically functional allowing for signal propagation around the lesion (Figures 2B through 2D).

To correlate the experimental results regarding PFA impact with the electric field generated in the hiPSC-CCSs between the 2 needle-shaped electrodes, we used a numeric model. As can be appreciated in Figure 2E, the resulting electric field is of elliptical shape, with the strongest field located around each electrode, while tapering down with distance. Consistent with previous reports,^36^ the electric field intensity and the culture area exposed to that field intensity could be fitted to an exponential function (Figure 2F, R^2^=0.99; *P*<0.001).

**Figure 3.**
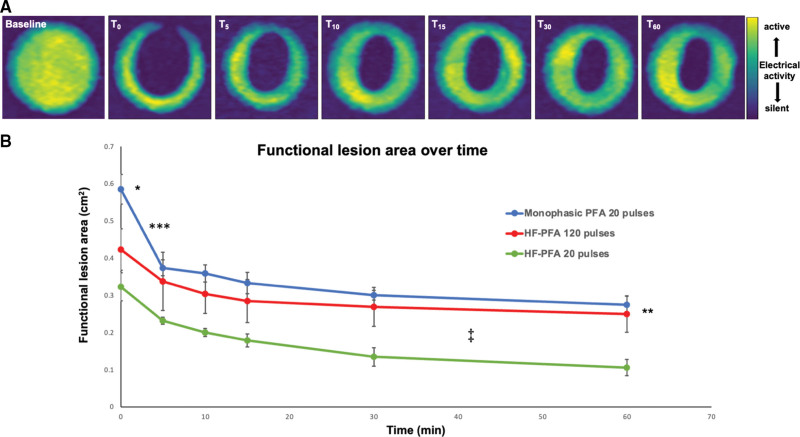
**Pulsed-field ablation (PFA) lesion-area changes over time. A**, Functional lesion mapping at 0, 5, 10, 15, 30, and 60-minutes post-PFA. Electrically active areas were identified based on frequency analysis in each pixel and are presented as color-coded maps (green/yellow – electrically active, dark blue – electrically silent). At baseline, the whole human-induced pluripotent stem cell (hiPSC)-derived cardiac circular cell sheets (hiPSC-CCS) is electrically active. Monophasic PFA (20 pulses) generated functional, electrically silent, lesions. Note the gradual decrease in lesion dimensions, reflecting recovery of electric activity with time (reversible electroporation). Also note that the central region remains electrically silent and the elliptical lesion shape consistent with the field-intensity distribution, which is strongest around each electrode and at the inter-electrode interface and decreases towards the periphery, when using 2 needle-shaped electrodes for PFA delivery. **B**, Lesion-area measurements over time following monophasic PFA (20 pulses, n=5 hiPSC-CCSs), compared with high-frequency (HF)-PFA (20 pulses, n=6 hiPSC-CCSs, and 120 pulses, n=5 hiPSC-CCSs). Note the initial lesion area and its decrease over time reflecting reversible electroporation kinetics; (1) Monophasic PFA generated the largest initial lesions (T_0_), followed by HF-PFA-120 pulses, and HF-PFA-20 pulses (0.59±0.04 cm^2^ for monophasic PFA 20 pulses vs 0.42±0.06 cm^2^ for HF-PFA 120 pulses and 0.32±0.04 cm^2^ for HF-PFA 20 pulses; *P<0.01 for each pairwise comparison). (2) Per single-pulse, monophasic PFA was more efficient than HF-PFA. Increasing HF-PFA pulse number significantly augmented lesion size, comparable to monophasic PFA at 60 minutes (**P<0.01 for monophasic PFA and HF-PFA-120 pulses vs HF-PFA-20 pulses. *P*=0.52 for monophasic PFA vs. HF-PFA-120 pulses). (3) A 2-stage recovery process is observed. The slope of reduction in the functional lesion area was significantly steeper with monophasic PFA (−0.042±0.003/min) as compared with HF-PFA-120 pulses (−0.018±0.003/min, ***P<0.01) or HF-PFA-20 pulses (−0.017±0.003/min, ***P<0.01). *P*=0.75 for differences between the HF-PFA protocols. From 10 to 60 minutes, recovery was markedly slower (10- to 20-fold slope reduction). (4) Increasing HF-PFA pulse number affected late, but not initial, reversible electroporation rate (‡*P*<0.01 for trace slope at 10–60 minutes comparing HF-PFA-120 pulses vs HF-PFA-20 pulses, and *P*=0.09 vs monophasic PFA).

Since electroporation may be reversible, we hypothesized that cells subjected to lower field intensities could potentially recover, regaining normal electric function. We, therefore, evaluated PFA-lesion dimensions over time. Sequential optical mapping images (Figure 3A) demonstrate dynamic, time-dependent recovery of cardiomyocyte electric activity within parts of the PFA lesions, reflecting reversible electroporation. The elliptical lesion shape was concordant with the field intensity distribution as was predicted by computational modeling (Figure 2E). Reversible electroporation and recovery of cardiomyocyte electric activity occurred where field intensities were lower. Conversely, cardiomyocytes located in the region surrounding each electrode, or in the inter-electrode interface, remained electrically silent over time, reflecting irreversible electroporation (Figure 3A). Cardiomyocyte recovery was characterized by 2 phases. Most of the recovery occurred during the initial phase, within 5 minutes following monophasic PFA, whereas the remaining functional recovery occurred at a significantly slower rate (Figure 3B).

### Comparing Standard Monophasic and High-Frequency PFA

Standard PFA protocols use square monophasic electric pulses. Apart from their local effects, such impulses can also be associated with remote nerve stimulation and muscle twitching. This obstacle may be overcome by utilizing modified high-frequency (HF) electroporation protocols, involving ultra-short bursts of biphasic pulses^8,34,37,38^ (Figure 1). Comparing the electrophysiological outcome following monophasic versus HF-PFA in the hiPSC-CCSs model, we found that per single pulse HF-PFA resulted in smaller functional lesions (Figure 3B).

Numerical modeling of the thresholds required for acute functional lesion formation suggested a 0.31±0.02 kV/cm threshold with monophasic PFA, as compared with 0.53±0.05 and 0.42±0.05 kV/cm thresholds with HF-PFA protocols (20 and 120 HF-PFA pulses, respectively; *P*<0.001 for monophasic PFA 20 pulses versus HF-PFA 20 pulses, and *P*≤0.005 versus HF-PFA 120 pulses).

Increasing HF-PFA pulse number (from 20 to 120 pulses, n=6 and n=5 different hiPSC-CCSs, respectively) resulted in significant lesion augmentation, achieving similar lesion dimensions as monophasic PFA (20 pulses, n=5 different hiPSC-CCSs) at 60-minute post-lesion deployment (0.27±0.02, 0.25±0.05, and 0.11±0.02 cm^2^ for monophasic PFA 20 pulses, HF-PFA 120 pulses, and HF-PFA 20 pulses, respectively. Figure 3B). Additionally, increasing HF-PFA pulses, from 20 to 120, had reduced lesion recovery within 60 minutes from 67±4% to 41±8% of the initial lesion area (*P*<0.001). This was mainly attributed to lower recovery rates between 10 and 60-minutes post-PFA (Figure 3B).

The lesion sustainability threshold at 60-minutes post-ablation, as derived from numerical modeling, was 0.6±0.04 kV/cm for monophasic PFA as compared with 1.03±0.08 and 0.65±0.1kV/cm for HF-PFA protocols (20 and 120 HF-PFA pulses, respectively; *P*<0.001 for monophasic PFA 20 pulses versus HF-PFA 20 pulses, and *P*=0.56 versus HF-PFA 120 pulses).

Lesion recovery kinetics also differed between the protocols. Although monophasic PFA generated the largest initial functional lesions (0.59±0.04 versus 0.42±0.06 cm^2^ for HF-PFA 120 pulses and 0.32±0.04 cm^2^ for HF-PFA 20 pulses; Figure 3B), the rapid cardiomyocyte recovery phase was also most prominent with this protocol. Hence, the slope of the rapid decrease in functional lesion area (within 5-minutes post-ablation) was significantly steeper with monophasic PFA (−0.042±0.003/min) as compared with HF-PFA-120 pulses (−0.018±0.003/min; *P*<0.01) or HF-PFA-20 pulses (−0.017±0.003/min; *P*<0.01).

### Lesion Sustainability at 24 Hours and Mechanisms of PFA-Mediated Cell Death

**Figure 4.**
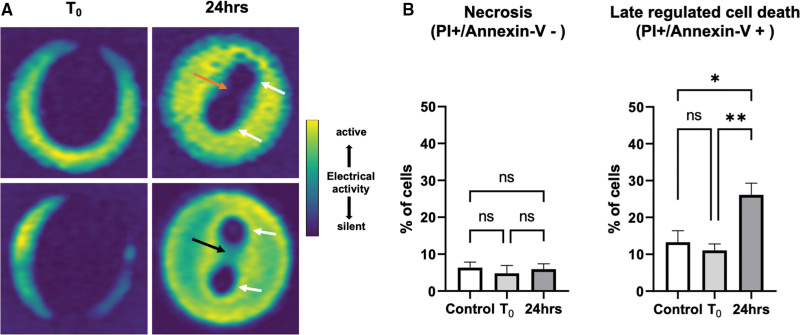
**Pulsed-field ablation (PFA) lesion sustainability and PFA-mediated cell-death mechanisms. A**, Mapping of functional lesions immediately (T_0_) and 24 hours following monophasic PFA (20 pulses). Electric activity recovery could be documented from T_0_ to 24-hour post-PFA, occurring mainly at the lesion periphery. The regions within the cultures that were in greatest proximity to the PFA-electrodes (white arrows) remained electrically silent at 24-hour post-PFA, reflecting irreversible electroporation. The middle-most portion of the lesion remained electrically silent in 70% of the cultures (top-panel, orange arrow), yet reversible electroporation and breach of the conduction block could be noted in this region in the remaining 30% (bottom-panel, black arrow). **B**, Flow-cytometry results comparing propidium iodine (PI) and Annexin-V staining in untreated control human-induced pluripotent stem cell (hiPSC)-derived cardiac circular cell sheets (hiPSC-CCSs; n=4) as compared with hiPSC-CCSs immediately (T_0_, n=4) and 24-hour (n=5) post-PFA. Accidental necrotic cell death (PI+/Annexin-V−, left) did not play a significant role, with no significant changes noted from T_0_ to 24 hour. Late regulated cell-death markers (PI+/Annexin-V+, right) significantly increased at 24-hour post-PFA, rising >2-fold (*P<0.05, **P<0.01). Importantly, there were no significant differences in Annexin-V and PI positive staining immediately following PFA (T_0_) as compared with untreated control hiPSC-CCSs.

Considering the possibility of ongoing cardiomyocyte recovery and reversible electroporation, we evaluated the hiPSC-CCSs conduction properties at 24-hour post-PFA. The presence of continuous electric silencing and persistent conduction blocks was documented, reflecting irreversible electroporation (Figure 4A). Comparing optical maps obtained immediately post-PFA (left-panels, T_0_) and 24 hours later (right panels), it is evident that further cardiomyocyte recovery had occurred over the period of hours. Hence, while cardiomyocytes in regions exposed to the highest field intensities (around each electrode and most of the interelectrode plane) were invariably subjected to irreversible electroporation, cardiomyocytes located further away exhibited reversible electroporation. Overall, lesion dimensions decreased significantly form 0.66±0.07 cm^2^ immediately following PFA to 0.15±0.04 cm^2^ at 24-hour post-PFA (n=10 different hiPSC-CCSs; *P*<0.01).

Numerical modeling revealed a 0.9±0.14 kV/cm electroporation irreversibility threshold at 24-hour post-PFA. Sustainable and complete functional lesions were documented at 24 hours within the inter-electrode interface in 7/10 (70%) of the cultures following monophasic PFA (20 pulses; Figure 4A, top panel). Interestingly, field intensity at the central portion of the inter-electrode plane ranged between 0.8 and 1 kV/cm based on the numerical modeling (Figure 2E). This allowed for functional recovery with a midline breach of the conduction block in the remaining 30% of the specimens (Figure 4A; bottom panel).

We next aimed to characterize the mechanisms of PFA-mediated cell death. It was previously suggested that regulated cell death plays an important role in irreversible electroporation.^1,7^ Flow cytometry was used to evaluate the proportion of PI and Annexin-V staining in dispersed cardiomyocytes derived from untreated control hiPSC-CCSs as compared with hiPSC-CCSs immediately (T_0_), and 24-hour post-PFA. Accidental necrosis (PI positive/Annexin-V negative staining) did not play a significant role neither immediately nor 24 hours following PFA (Figure 4B, left panel). On the contrary, late-phase regulated cell-death markers (PI positive/Annexin-V positive staining) significantly increased (rising >2-fold) at 24-hour post-PFA as compared with T_0_ (Figure 4B; right panel). Importantly, there were no significant differences in Annexin-V and PI positive staining immediately following PFA (T_0_) as compared with untreated control hiPSC-CCSs.

### Ca^2+^ and Electroporation Sensitization

**Figure 5.**
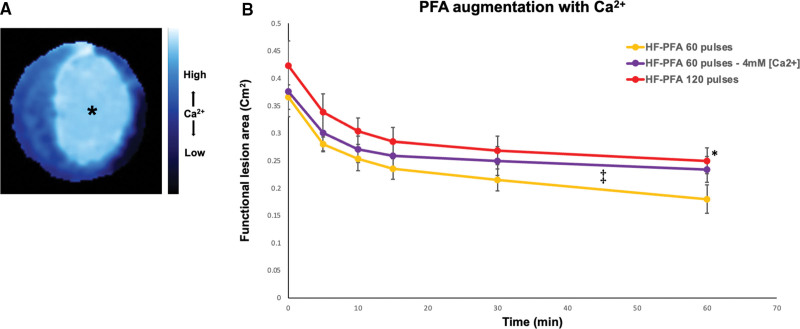
**Role of Ca** ^2^**^+^ in pulsed-field ablation (PFA). A**, Ca^2+^-imaging of Fluo4-loaded human-induced pluripotent stem cell (hiPSC)-derived cardiac circular cell sheets (hiPSC-CCS) following PFA showing increased intracellular Ca^2+^-levels within the PFA lesion (asterisk). **B**, Lesion-area measurements over time following high-frequency (HF)-PFA, comparing standard and increased extracellular Ca^2+^ concentrations (tyrode’s containing 1.8 vs 4 mM CaCl_2_, respectively). Ca^2+^ enriched HF-PFA (60 pulses) augmented lesion size at 60 minutes, an effect comparable to doubling the pulse number under standard Ca^2+^ levels (*P<0.01 for HF-PFA-60 pulses [4 mM Ca^2+^] vs HF-PFA-60 pulses, and *P*=0.7 vs HF-PFA-120 pulses). This effect was related to attenuated late-stage recovery, manifested by a significant decrease in recovery rate at 10 to 60 minUTES (‡*P*<0.01 for trace slope comparing HF-PFA-60 pulses-[4 mM Ca^2+^] vs HF-PFA-60 pulses, and *P*=0.53 vs. HF-PFA-120 pulses).

As noted above, PFA lesions were relatively depolarized and electrically silent. Since Ca^2+^ plays an important role in cardiomyocyte electrophysiology, and since membrane integrity is crucial for such normal function, we performed Ca^2+^ imaging of Fluo-4 loaded hiPSC-CCSs following PFA and found increased Ca^2+^ levels within PFA lesions (Figure 5A), consistent with recent reports.^39^

It was previously suggested that increasing cytoplasmatic Ca^2+^ plays an important role in PFA-mediated cytolysis,^5–7^ and that intracellular Ca^2+^ levels significantly increase before electroporation induced cell death.^39^ We hypothesized that electroporation sensitization and augmentation could be achieved by increasing cardiomyocyte Ca^2+^ entry and concentration following electroporation, through elevation of extracellular Ca^2+^ levels. To test this hypothesis, we compared PFA lesion dimensions under standard conditions and in a Ca^2+^-rich environment (Tyrode containing 4 mM CaCl_2_). Interestingly, increasing Ca^2+^ levels mostly impacted later stages of cellular recovery, significantly augmenting lesion size by almost 30% (which was comparable to doubling the pulse number with standard Ca^2+^ concentrations, Figure 5B).

### Arrhythmia Targeting With PFA

**Figure 6.**
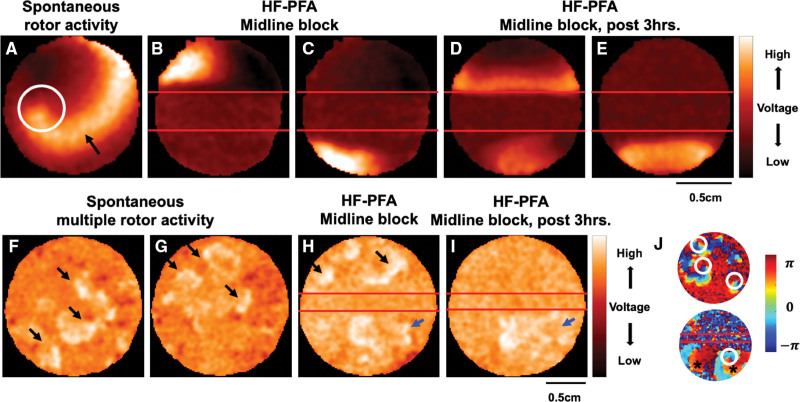
**Arrhythmia targeting with pulsed-field ablation (PFA).** Optical mapping of arrhythmogenic activity in human-induced pluripotent stem cell (hiPSC)-derived cardiac circular cell sheets (hiPSC-CCSs). **A**, A single rotor (rotor-core encircled) and its wave front (arrow), rotating clockwise. **B** through **E**, The same hiPSC-CCS following application of ablation lesions at the mid-horizontal plane (red lines) using a point-by-point HF-PFA approach (50 pulses repeatedly deployed in 5 consecutive locations). Ablation eliminated the rotor-core while generating a midline-block isolating the 2 culture portions. Asynchronous spontaneous electric activity is evident in the upper (**B**) and lower (**C**) culture portions. **D** through **E**, Persistent midline conduction block, 3 hours post-PFA. Pacing the culture from the upper (**D**) or lower (**E**) portions confirms a bidirectional conduction block. **F** through **G**, Optical mapping demonstrating multiple spontaneous rotors in the hiPSC-CCS. **H** through **I**, Optical mapping of the same hiPSC-CCS immediately (**H**) and 3 hours (**I**) following creation of a linear midline lesion (red-lines) using a point-by-point HF-PFA approach (50 pulses repeatedly deployed in 5 consecutive locations). Ablation eliminated arrhythmic activity within the treated area. The remaining arrhythmic substrate was isolated and confined by the linear lesion. **J**, Phase-mapping analysis of the same culture (rotor cores encircled). Following HF-PFA (bottom-panel), the lower right rotor (blue arrows in **H** and **I**) is mapped, rotating clockwise. Its propagating wave fronts (asterisks) are now blocked at midline.

Different forms of reentry underlie most atrial and ventricular arrhythmias. Rotors, manifested as spiral- or scroll-waves within the tissue, are a unique recently recognized form of reentry,^40^ which can also be induced and studied in the hiPSC-CCS model.^27–30^ To evaluate arrhythmia targeting by PFA, we used hiPSC-CCSs plated at lower cell-density either focally or diffusely, facilitating the spontaneous emergence of single or multiple rotors (Figure 6A through 6J). PFA was delivered in a linear manner (using a point-by-point approach) to generate a continuous line of conduction block, spanning the entire diameter of the hiPSC-CCSs. The resulting PFA lesions eliminated arrhythmic rotors (or any electric activity) within the ablated area (Figure 6B through 6E, 6H, and 6I) and electrically isolated the opposing portions of the hiPSC-CCSs, creating a bi-directional block (Figure 6B through 6E, and 6H through 6J). In the case of a single spiral wave using the entire culture area (Video S3), such ablation was sufficient to terminate the arrhythmic activity (Figure 6B and 6E, Video S4). In the case of multiple rotors, ablation terminated arrhythmogenicity locally, and allowed for the isolation of the remaining arrhythmic activity to confined regions (Figures 6H through 6J).

## DISCUSSION

In this work, we established and characterized a novel functional hiPSC-based in vitro model to study electroporation mechanisms and impact in human cardiomyocytes, while focusing on tissue electrophysiological characteristics. Using this model, we were able to evaluate multiple fundamental aspects of cardiac PFA, gaining insights into its mechanism of action.

Our main findings include: (1) PFA resulted in the formation of highly localized lesions, exhibiting both reversible and irreversible electroporation; (2) depending on the protocol, reversible electroporation could involve up to 67% of the initial lesion area at 60-minutes post-PFA; (3) significant reversible electroporation was documented within the first 5 to 15-minutes post-PFA; (4) per single pulse, HF-PFA was less efficacious than monophasic PFA, generating smaller lesions; (5) increasing pulse-number augmented lesion area and decreased reversible electroporation; (6) prolonged lesion sustainability (irreversible electroporation) was documented at 24-hours post-PFA, with an irreversibility threshold of ≈0.9 kV/cm; (7) regulated cell death plays an important role following PFA; (8) electroporation sensitization was achieved by increasing extracellular Ca^2+^ levels during PFA; (9) PFA was effective in terminating arrhythmic activity and isolating arrhythmogenic substrate within the hiPSC-CCSs by generating sustainable lines of conduction block; (10) PFA effects stem from a combination of cellular electrophysiological changes (including membrane depolarization and increased intracellular Ca^2+^), cellular damage, and cell death.

We were able to study reversible and irreversible electroporation temporal kinetics and characterize how they were influenced by protocol modifications. Significant cardiomyocyte recovery was observed following PFA, with resumption of electric activity in 40% to 67% of the initial functional lesion within 1-hour post-PFA. Consistent with previous reports,^33,34,38^ protocol modifications could influence the extent of reversible electroporation, with a tight spatial correlation between the expected distribution of the electric field intensity and the eventual occurrence of irreversible electroporation. These observations have important clinical consequences, as the immediate development of localized electric silencing and functional conduction blocks does not necessarily mean long-term persistence of these lesions. Our findings also offer time frames that might be potentially relevant for the clinical setup with regards to reassessing the electric activity following initial PFA delivery.

Traditional electroporation protocols incorporated square monophasic pulses, typically of 50 to 100 μsec duration. Such impulses may also be associated with remote nerve stimulation and muscle twitching, limiting their clinical use^37^ due to patient movements during ablation, patient-discomfort, or the requirement for general anesthesia and muscle relaxation.^8,10^ One approach to overcome this obstacle is using modified high-frequency electroporation protocols involving ultra-short bursts of biphasic pulses (<10 μsec/phase).^8,34,37,38^ Ongoing efforts are being directed at comparing such different protocols in vitro and in vivo.^34^ Here, by comparing monophasic and HF-PFA protocols (Figure 1) we found that, per single pulse, HF-PFA was less efficacious generating smaller functional lesions and requiring higher field-intensity thresholds for lesion formation and sustainability. This may be related to decreased total time at which the trans-membrane potential is above irreversibility threshold, as well as with the specific HF-PFA pulse settings.^37^

Increasing HF-PFA pulse number, thereby increasing the cumulative cardiomyocyte exposure to electric energy, had reduced the level of reversible electroporation and enabled achieving lesions comparable to monophasic PFA in size and field intensity threshold, at a pulse ratio of ≈1:6. These findings are consistent with previous in vivo studies,^34,41^ and imply that HF-PFA protocols may achieve comparable results to monophasic-PFA, maintaining the significant advantage of reduced muscle stimulation. Interestingly, monophasic-PFA generated the largest initial lesions (as also reflected by the significantly lower threshold for acute lesion formation) while also characterized by the most prominent rapid recovery component, with significant reversible electroporation occurring within 5-minutes post-PFA, similar to previous reports.^39^ In contrast, HF-PFA protocols were associated with smaller initial lesions, but also a significantly slower initial recovery. Hence, cardiomyocyte recovery (and especially early phase reversible electroporation) is different between monophasic and HF-PFA.

Several clinically available PFA systems allow for biphasic- and HF-PFA. Nevertheless, such optimized biphasic protocols although used clinically, remain not fully disclosed by the industry, and each manufacturer offers different pulse setups and protocols which are modifiable by the operating clinician only to a limited extent. Hence, HF-PFA protocol advantages, disadvantages and temporal kinetics should be further studied, characterized, and standardized. Importantly, full disclosure of clinically used PFA protocols is needed, as even minor protocol changes may lead to significant differences in PFA outcomes.

Assessing longer term lesion sustainability, persistent conduction blocks were documented at 24-hour post-PFA, reflecting irreversible electroporation with an irreversibility threshold of ≈0.9 kV/cm. This is consistent with previous in vivo and in vitro reports, including recent studies utilizing hiPSC-CMs.^13,14,33,34,37,41^ In regions of the culture subjected to lower field intensities, a delayed recovery of electric activity could be detected. These findings further emphasize that initial electric silencing following PFA does not necessarily translate into durable lesions, and that recovery of electric activity may occur over the course of hours/days. Considering recent reports of 1-year clinical PFA outcomes,^16,17^ our findings emphasize the importance of further research into identifying robust predictors of long-term lesion persistence during acute PFA application.

We demonstrated that pure necrotic cell death did not play a significant role nor immediately, neither late following PFA. Regulated cell-death markers, on the contrary, were significantly increased (>2-fold) at 24-hour post-PFA. These findings reflect the ongoing nature of the cumulative electroporation-induced cellular damage, leading to time dependent, gradual, and regulated cell death. Although cell-viability assays often rely on membrane integrity which may potentially limit their relevance in the context of electroporation, we did not observe significant differences in Annexin-V or PI staining comparing untreated control hiPSC-CCSs with hiPSC-CCSs collected immediately post-PFA. This excludes a potential bias attributed to membrane electroporation per se.

Our results highlight the different mechanisms that may underlie PFA electrophysiological effects. Although the late PFA effect probably stems from irreversible electroporation and regulated cell death,^7^ the acute effects may stem from a combination of cellular electrophysiological changes and cardiomyocyte injury induced by membrane damage. Voltage- and calcium-based optical imaging showed that the acute PFA lesions were characterized by increased resting membrane potential and intracellular Ca^2+^ levels, which may lead to electric silencing through Na^+^-channel inactivation.

We also demonstrated that by increasing extracellular Ca^2+^ levels, we could augment PFA lesions to an extent comparable with doubling the pulse number under standard Ca^2+^ concentrations. Interestingly, PFA augmentation with Ca^2+^ occurred mainly during the later stages postablation and not immediately, which suggests a role for Ca^2+^ in the gradual and regulated cellular damage induced by PFA. This PFA sensitization approach may allow for further clinical protocol optimization, for instance by irrigating the catheter tip with a Ca^2+^ rich solution during ablation, thereby increasing its efficacy.

Using the hiPSC-CCS model, we demonstrated the effective targeting and elimination of single or multiple arrhythmic rotors with PFA, through the formation of sustainable bidirectional linear conduction blocks. Such continuous lesions were also able to isolate arrhythmogenic substrates within the cultures. Nevertheless, it is noteworthy that the role of rotors in clinical atrial fibrillation remains controversial and the interpretation of these in vitro findings to clinical arrhythmias should be done with caution.

Finally, the generation of targeted lesions sized ≤0.7 cm^2^, and the ability to affect only the midline portion of ≤3 cm^2^ cultures, underscore the immense potential of PFA in targeting cardiomyocytes at a desired location with high precision. This is also supported by the exponential relationship between the field intensity and the culture area exposed to that intensity as revealed by numerical modeling.

Our study is not without limitations. We were unable to exclude significant thermal effects following PFA, which could be present especially in the immediate electrode vicinity, yet the protocols used were previously shown to cause only minor temperature changes.^34^ Although collecting the supernatant medium in flow-cytometry experiments to avoid loss of detached cells, we could not exclude some loss of fragmented cell debris during centrifugation, washes, and acquired data gating, which could potentially underestimate necrotic cell death. Additionally, characterizing the exact programmed/regulated cell-death pathway (apoptosis, necroptosis, etc), and the role of the immune system in cell death, was beyond the scope of this work. We evaluated lesion sustainability and regulated cell death within 24-hour post-PFA. As additional cellular recovery or ongoing cellular damage may potentially occur over the course of days, further studies are required assessing PFA lesions dynamics and durability over the course of days to weeks. Additionally, the irreversibility thresholds as derived from numerical models are influenced by multiple parameters affecting electroporation (such as voltage, pulse number, pulse shape, pulse duration, pulse polarity, and the electrode setup). Subsequently, such thresholds are protocol specific and may vary significantly between different protocols. Further research is warranted for the development of statistical models, accurately predicting the probability of irreversible electroporation as influenced by PFA protocols or tissue composition.

Further research is also warranted into the role of specific currents, including Na^+^, K^+^, and Ca^2+^ in PFA-mediated electric silencing, cellular recovery, and cell death. Interestingly, we noted occasional and transient increase in automaticity at the border zone of the PFA lesions, during cardiomyocyte recovery. This implies that PFA lesions may be transiently proarrhythmic, which also requires further investigation.

In summary, our study provides novel insights into PFA temporal and electrophysiological characteristics and facilitates electroporation protocol optimization, screening for potential PFA-sensitizers, and studying the mechanistic nature of its antiarrhythmic properties. It provides further support that hiPSC-based models may be used to study multiple aspects of PFA and should facilitate future bedside to bench and back studies in this rapidly evolving field of cardiac electrophysiology.

## ARTICLE INFORMATION

### Sources of Funding

This work was partially funded by research grants from the Alrov Fund, the Gassner Fund for Medical Research, the Crown Foundation, the Israel Ministry of Science and Technology (grant #100157604), and the European Research Council (ERC-2017-COG-773181-iPS-ChOp-AF).

### Disclosures

None.

### Supplemental Material

Videos S1–S4

## Supplementary Material

## References

[1] Maor E Sugrue A Witt C Vaidya VR DeSimone C Asirvatham SJ Kapa S. Pulsed electric fields for cardiac ablation and beyond: a state-of-the-art review. Heart Rhythm. 2019;16:1112–1120. doi: 10.1016/j.hrthm.2019.01.012 30641148 10.1016/j.hrthm.2019.01.012

[2] Yuyun MF Stafford PJ Sandilands AJ Samani NJ Ng GA. The impact of power output during percutaneous catheter radiofrequency ablation for atrial fibrillation on efficacy and safety outcomes: a systematic review. J Cardiovasc Electrophysiol. 2013;24:1216–1223. doi: 10.1111/jce.12206 23890323 10.1111/jce.12206

[3] Cappato R Calkins H Chen SA Davies W Iesaka Y Kalman J Kim YH Klein G Natale A Packer D. Prevalence and causes of fatal outcome in catheter ablation of atrial fibrillation. J Am Coll Cardiol. 2009;53:1798–1803. doi: 10.1016/j.jacc.2009.02.022 19422987 10.1016/j.jacc.2009.02.022

[4] Matsuki N Ishikawa T Imai Y Yamaguchi T. Low voltage pulses can induce apoptosis. Cancer Lett. 2008;269:93–100. doi: 10.1016/j.canlet.2008.04.019 18504072 10.1016/j.canlet.2008.04.019

[5] Frandsen SK Gissel H Hojman P Tramm T Eriksen J Gehl J. Direct therapeutic applications of calcium electroporation to effectively induce tumor necrosis. Cancer Res. 2012;72:1336–1341. doi: 10.1158/0008-5472.CAN-11-3782 22282658 10.1158/0008-5472.CAN-11-3782

[6] Hansen EL Sozer EB Romeo S Frandsen SK Vernier PT Gehl J. Dose-Dependent ATP depletion and cancer cell death following calcium electroporation, relative effect of calcium concentration and electric field strength. PLoS One. 2015;10:e0122973. doi: 10.1371/journal.pone.0122973 25853661 10.1371/journal.pone.0122973 PMC4390219

[7] Batista Napotnik T Polajžer T Miklavčič D. Cell death due to electroporation – a review. Bioelectrochemistry. 2021;141:107871. doi: 10.1016/j.bioelechem.2021.107871 34147013 10.1016/j.bioelechem.2021.107871

[8] Reddy VY Koruth J Jais P Petru J Timko F Skalsky I Hebeler R Labrousse L Barandon L Kralovec S. Ablation of atrial fibrillation with pulsed electric fields. JACC Clin Electrophysiol. 2018;4:987–995. doi: 10.1016/j.jacep.2018.04.005 30139499 10.1016/j.jacep.2018.04.005

[9] Wittkampf FH van Driel VJ van Wessel H Vink A Hof IE Gründeman PF Hauer RNW Loh P. Feasibility of electroporation for the creation of pulmonary vein ostial lesions. J Cardiovasc Electrophysiol. 2011;22:302–309. doi: 10.1111/j.1540-8167.2010.01863.x 20653809 10.1111/j.1540-8167.2010.01863.x

[10] Loh P Es R van Groen MHA Neven K Kassenberg W Wittkampf FHM Doevendans PA. Pulmonary vein isolation with single pulse irreversible electroporation. Circ Arrhythm Electrophysiol. 2020;13:e008192. doi: 10.1161/CIRCEP.119.008192 32898450 10.1161/CIRCEP.119.008192

[11] Witt CM Sugrue A Padmanabhan D Vaidya V Gruba S Rohl J DeSimone CV Killu AM Naksuk N Pederson J. Intrapulmonary vein ablation without stenosis: a novel balloon-based direct current electroporation approach. J Am Heart Assoc. 2018;7:e009575. doi: 10.1161/JAHA.118.009575 29987121 10.1161/JAHA.118.009575 PMC6064854

[12] Lavee J Onik G Mikus P Rubinsky B. A novel nonthermal energy source for surgical epicardial atrial ablation: irreversible electroporation. Heart Surg Forum. 2007;10:E162–E167. doi: 10.1532/HSF98.20061202 17597044 10.1532/HSF98.20061202

[13] Casciola M Keck D Feaster TK Blinova K. Human cardiomyocytes are more susceptible to irreversible electroporation by pulsed electric field than human esophageal cells. Physiol Rep. 2022;10:e15493. doi: 10.14814/phy2.15493 36301726 10.14814/phy2.15493 PMC9612150

[14] Moshkovits Y Grynberg D Heller E Maizels L Maor E. Differential effect of high-frequency electroporation on myocardium vs. non-myocardial tissues. Europace. 2023;25:748–755. doi: 10.1093/europace/euac191 36305566 10.1093/europace/euac191 PMC9935033

[15] Reddy VY Anic A Koruth J Petru J Funasako M Minami K Breskovic T Sikiric I Dukkipati SR Kawamura I. Pulsed field ablation in patients with persistent atrial fibrillation. J Am Coll Cardiol. 2020;76:1068–1080. doi: 10.1016/j.jacc.2020.07.007 32854842 10.1016/j.jacc.2020.07.007

[16] Reddy VY Turagam MK Neuzil P Schmidt B Reichlin T Neven K Metzner A Hansen J Blaauw Y Maury P. Safety and effectiveness of pulsed field ablation to treat atrial fibrillation: one-year outcomes from the MANIFEST-PF registry. 2023;148:35. doi: 10.1161/CIRCULATIONAHA.123.064959 10.1161/CIRCULATIONAHA.123.064959 37199171

[17] Reddy VY Dukkipati SR Neuzil P Anic A Petru J Funasako M Cochet H Minami K Breskovic T Sikiric I. Pulsed field ablation of paroxysmal atrial fibrillation: 1-year outcomes of IMPULSE, PEFCAT, and PEFCAT II. JACC Clin Electrophysiol. 2021;7:614–627. doi: 10.1016/j.jacep.2021.02.014 33933412 10.1016/j.jacep.2021.02.014

[18] Ladejobi A Christopoulos G Tan N Ladas TP Tri J Zyl M van Yasin O Sugrue A Khabsa M Uecker DR. Effects of pulsed electric fields on the coronary arteries in swine. Circ Arrhythm Electrophysiol. 2022;15:e010668. doi: 10.1161/CIRCEP.121.010668 36194538 10.1161/CIRCEP.121.010668

[19] Reddy VY Petru J Funasako M Kopriva K Hala P Chovanec M Janotka M Kralovec S Neuzil P. Coronary arterial spasm during pulsed field ablation to treat atrial fibrillation. Circulation. 2022;146:1808–1819. doi: 10.1161/CIRCULATIONAHA.122.061497 36134574 10.1161/CIRCULATIONAHA.122.061497

[20] Zwi L Caspi O Arbel G Huber I Gepstein A Park IH Gepstein L. Cardiomyocyte differentiation of human induced pluripotent stem cells. Circulation. 2009;120:1513–1523. doi: 10.1161/CIRCULATIONAHA.109.868885 19786631 10.1161/CIRCULATIONAHA.109.868885

[21] Zhang J Wilson GF Soerens AG Koonce CH Yu J Palecek SP Thomson JA Kamp TJ. Functional cardiomyocytes derived from human induced pluripotent stem cells. Circ Res. 2009;104:e30–e41. doi: 10.1161/CIRCRESAHA.108.192237 19213953 10.1161/CIRCRESAHA.108.192237 PMC2741334

[22] Itzhaki I Maizels L Huber I Gepstein A Arbel G Caspi O Miller L Belhassen B Nof E Glikson M. Modeling of catecholaminergic polymorphic ventricular tachycardia with patient-specific human-induced pluripotent stem cells. J Am Coll Cardiol. 2012;60:990–1000. doi: 10.1016/j.jacc.2012.02.066 22749309 10.1016/j.jacc.2012.02.066

[23] Itzhaki I Maizels L Huber I Zwi-Dantsis L Caspi O Winterstern A Feldman O Gepstein A Arbel G Hammerman H. Modelling the long QT syndrome with induced pluripotent stem cells. Nature. 2011;471:225–229. doi: 10.1038/nature09747 21240260 10.1038/nature09747

[24] Maizels L Huber I Arbel G Tijsen AJ Gepstein A Khoury A Gepstein L. Patient-specific drug screening using a human induced pluripotent stem cell model of catecholaminergic polymorphic ventricular tachycardia type 2. Circ Arrhythm Electrophysiol. 2017;10:e004725. doi: 10.1161/CIRCEP.116.004725 28630169 10.1161/CIRCEP.116.004725

[25] Moretti A Bellin M Welling A Jung CB Lam JT Bott-Flügel L Dorn T Goedel A Höhnke C Hofmann F. Patient-specific induced pluripotent stem-cell models for long-QT syndrome. N Engl J Med. 2010;363:1397–1409. doi: 10.1056/NEJMoa0908679 20660394 10.1056/NEJMoa0908679

[26] Matsa E Ahrens JH Wu JC. Human induced pluripotent stem cells as a platform for personalized and precision cardiovascular medicine. Physiol Rev. 2016;96:1093–1126. doi: 10.1152/physrev.00036.2015 27335446 10.1152/physrev.00036.2015 PMC6345246

[27] Shaheen N Shiti A Huber I Shinnawi R Arbel G Gepstein A Setter N Goldfracht I Gruber A Chorna SV. Human induced pluripotent stem cell-derived cardiac cell sheets expressing genetically encoded voltage indicator for pharmacological and arrhythmia studies. Stem Cell Rep. 2018;10:1879–1894. doi: 10.1016/j.stemcr.2018.04.006 10.1016/j.stemcr.2018.04.006 PMC5989818 29754959

[28] Shinnawi R Shaheen N Huber I Shiti A Arbel G Gepstein A Ballan N Setter N Tijsen AJ Borggrefe M. Modeling reentry in the short QT syndrome with human-induced pluripotent stem cell–derived cardiac cell sheets. J Am Coll Cardiol. 2019;73:2310–2324. doi: 10.1016/j.jacc.2019.02.055 31072576 10.1016/j.jacc.2019.02.055

[29] Shinnawi R Huber I Maizels L Shaheen N Gepstein A Arbel G Tijsen AJ Gepstein L. Monitoring human-induced pluripotent stem cell-derived cardiomyocytes with genetically encoded calcium and voltage fluorescent reporters. Stem Cell Rep. 2015;5:582–596. doi: 10.1016/j.stemcr.2015.08.009 10.1016/j.stemcr.2015.08.009 PMC4624957 26372632

[30] Feola I Volkers L Majumder R Teplenin A Schalij MJ Panfilov AV de Vries AAF Pijnappels DA. Localized optogenetic targeting of rotors in atrial cardiomyocyte monolayers. Circ Arrhythm Electrophysiol. 2017;10:e005591. doi: 10.1161/CIRCEP.117.005591 29097406 10.1161/CIRCEP.117.005591

[31] Karakikes I Ameen M Termglinchan V Wu JC. Human induced pluripotent stem cell–derived cardiomyocytes. Circ Res. 2015;117:80–88. doi: 10.1161/CIRCRESAHA.117.305365 26089365 10.1161/CIRCRESAHA.117.305365 PMC4546707

[32] Onódi Z Visnovitz T Kiss B Hambalkó S Koncz A Ágg B Váradi B Tóth V Nagy RN Gergely TG. Systematic transcriptomic and phenotypic characterization of human and murine cardiac myocyte cell lines and primary cardiomyocytes reveals serious limitations and low resemblances to adult cardiac phenotype. J Mol Cell Cardiol. 2022;165:19–30. doi: 10.1016/j.yjmcc.2021.12.007 34959166 10.1016/j.yjmcc.2021.12.007

[33] Casciola M Feaster TK Caiola MJ Keck D Blinova K. Human in vitro assay for irreversible electroporation cardiac ablation. Front Physiol. 2023;13:1064168. doi: 10.3389/fphys.2022.1064168 36699682 10.3389/fphys.2022.1064168 PMC9869257

[34] Heller E Garcia-Sanchez T Moshkovits Y Rabinovici R Grynberg D Segev A Asirvatham SJ Ivorra A Maor E. Comparing high-frequency with monophasic electroporation protocols in an in vivo beating heart model. JACC Clin Electrophysiol. 2021;7:959–964. doi: 10.1016/j.jacep.2021.05.003 34217666 10.1016/j.jacep.2021.05.003

[35] O’Shea C Holmes AP Yu TY Winter J Wells SP Correia J Boukens BJ Groot JR De Chu GS Li X. ElectroMap: high-throughput open-source software for analysis and mapping of cardiac electrophysiology. Sci Rep. 2019;9:1389. doi: 10.1038/s41598-018-38263-2 30718782 10.1038/s41598-018-38263-2 PMC6362081

[36] Sharabi S Last D Guez D Daniels D Hjouj MI Salomon S Maor E Mardor Y. Dynamic effects of point source electroporation on the rat brain tissue. Bioelectrochemistry. 2014;99:30–39. doi: 10.1016/j.bioelechem.2014.06.001 24976141 10.1016/j.bioelechem.2014.06.001

[37] Arena CB Sano MB Rossmeisl JH Caldwell JL Garcia PA Rylander MN Davalos RV. High-frequency irreversible electroporation (H-FIRE) for non-thermal ablation without muscle contraction. Biomed Eng Online. 2011;10:102. doi: 10.1186/1475-925X-10-102 22104372 10.1186/1475-925X-10-102 PMC3258292

[38] Miklovic T Latouche EL DeWitt MR Davalos RV Sano MB. A comprehensive characterization of parameters affecting high-frequency irreversible electroporation lesions. Ann Biomed Eng. 2017;45:2524–2534. doi: 10.1007/s10439-017-1889-2 28721494 10.1007/s10439-017-1889-2

[39] Chaigne S Sigg DC Stewart MT Hocini M Batista Napotnik T Miklavčič D Bernus O Benoist D. Reversible and irreversible effects of electroporation on contractility and calcium homeostasis in isolated cardiac ventricular myocytes. Circ Arrhythm Electrophysiol. 2022;15:e011131. doi: 10.1161/CIRCEP.122.011131 36306333 10.1161/CIRCEP.122.011131 PMC9665944

[40] Pandit SV Jalife J. Rotors and the dynamics of cardiac fibrillation. Circ Res. 2013;112:849–862. doi: 10.1161/CIRCRESAHA.111.300158 23449547 10.1161/CIRCRESAHA.111.300158 PMC3650644

[41] García-Sánchez T Amorós-Figueras G Jorge E Campos MC Maor E Guerra JM Ivorra A. Parametric study of pulsed field ablation with biphasic waveforms in an in vivo heart model: the role of frequency. Circ Arrhythm Electrophysiol. 2022;15:e010992. doi: 10.1161/CIRCEP.122.010992 36178752 10.1161/CIRCEP.122.010992

